# Factors associated with smoking susceptibility among high school students in western Spain

**DOI:** 10.1038/s41598-021-81723-5

**Published:** 2021-01-21

**Authors:** E. Santano-Mogena, C. Franco-Antonio, S. Chimento-Díaz, S. Rico-Martín, S. Cordovilla-Guardia

**Affiliations:** 1 Nursing Department, Nursing and Occupational Therapy College, University of Extremadura, Cáceres, Spain; 2 Health and Care Research Group (GISyC), University of Extremadura, Cáceres, Spain; 3 Computer Systems Engineering and Telematics Department, Polytechnic School of Cáceres, University of Extremadura, Cáceres, Spain

**Keywords:** Health care, Public health

## Abstract

The Expanded Susceptibility to Smoking Index (ESSI) is based on the combination of susceptibility to smoking and curiosity about smoking. The ESSI can identify young people who are at risk of starting to smoke cigarettes and related products. The objective of this study was to analyse the ESSI results and to examine factors associated with ESSI scores in students between 12 and 16 years of age. Sociodemographic, social/environmental and personal variables were analysed, and the ESSI value was determined for non-smoking students recruited from three schools in western Spain. Regression models were used to examine the factors associated with smoking for the entire sample and the factors associated with ESSI scores in the non-smoking population. Of the 377 participants who were analysed, 20.4% were smokers. Among the non-smokers, 53.5% and 55.3% presented medium–high ESSI scores for cigarettes and e-cigarettes, respectively, and 39.8% presented medium–high ESSI scores for hookah use. A higher ESSI score was associated with greater exposure to people smoking in the home, having more friends who smoke, alcohol consumption, and a higher impulsivity scale score. These findings reinforce the importance of reducing peer pressure and suggest the important role of resolve under conditions of positive affect on reducing impulsivity. Approaches based on self-efficacy could be addressed in preventive programmes developed in educational settings.

## Introduction

The prevalence of cigarette smoking among adolescents and young people continues to be high. According to a study conducted in 35 European countries in 2015^1^, 21% of adolescents used tobacco regularly (in the previous 30 days). However, the estimates show important variations among countries. Recently, Spain has seen a reduction in the prevalence of regular cigarette use among young people aged 11 to 18 years, from 8.9% in 2010 to 4.5% in 2018^2^. When the youngest smokers are excluded and the age range is narrowed to 14–18 years, we see that the rate of cigarette smoking increased to 9.8% in 2018, although this value was 2.5% less than in 2010^3^. Despite this reduction in cigarette smoking among adolescents, smoking remains a major health problem^4^ due to the emergence of other related products, such as electronic cigarettes (e-cigarettes) and hookahs, whose use has increased in recent years^5,6^.

Electronic cigarettes, or electronic nicotine delivery systems (ENDS), provide nicotine in the form of an aerosol or vapor^5^. ENDS seem to have a special appeal among adolescents and young people^7^, especially those who have not started smoking^8^. Since these products were first commercialized in 2006^9^, their use has been increasing, and they are currently the most used smoking product by young people in the United States, ranking above tobacco cigarettes^10^. By 2017, one in four young people (aged 15–24 years) in Europe had tried ENDS^11^. In our country (Spain), 14.9% of adolescents had smoked e-cigarettes in the previous month^3^. In comparison, hookahs (water pipes) vaporize tobacco and other products by combustion; the smoke generated travels through the water before it is inhaled^6^. Although this is not a new system for smoking, it has become trendy, and its popularity has continued to increase in recent years, especially among adolescents and young people^12^. According to the 2017 Eurobarometer, 28% of young people between 15 and 24 years had tried a hookah, and 2% used one monthly^11^. In Spain, the data on hookah use are limited; according to one study conducted among high school students, 13% used a hookah on a weekly basis^13^.

Adolescence is a crucial developmental period^14,15^ characterized by engagement in risky behaviours^16^, including tobacco consumption. In fact, 90% of smokers start smoking before the age of 18 years^17^. Prior to the first smoking attempt, some areas of cognition, such as curiosity and susceptibility to smoking (SS), are developed that increase the probability of initiating tobacco use^18,19^. SS is defined as the lack of a specific and firm commitment to not smoke^20,21^. It reflects an individual's decision regarding smoking and influences the transition from non-smoker to smoker status. It is assessed through intention, self-efficacy and the influence of the social environment^21^. SS, which begins to develop in childhood^22^, has proven to be a good predictor of whether an individual will start to smoke^18,23^. In the adolescent population, several longitudinal studies have concluded that susceptibility predicts subsequent initiation^22–24^ and therefore can be used to identify adolescents who are at risk of starting to smoke cigarettes^18,23^ and related products, such as e-cigarettes and hookahs^22,24^.

Along with SS, curiosity^25^ has been shown to have a high value for predicting the initiation of smoking^18,21^. Based on the combination of these two predictors, a new measure has been developed, the Expanded Susceptibility to Smoking Index (ESSI)^18,19,23^. This measure improves the prediction of smoking initiation and allows for more precise identification of adolescents who are at risk of starting to smoke^19^.

Certain sociodemographic, social and personal factors can influence SS, including age, gender, parental educational level and the socioeconomic level of the family unit^26^. The behaviour of different models in the social environment^27^, the social pressures exerted by the peer group and the number of offers to smoke^28^, the search for rewarding stimuli^29^, poor impulse control^30^, states of negative affect^31^, and low risk perception and beliefs about the harms produced by smoking^32–34^ have been associated with changes in intention and self-efficacy, which could modify the SS.

E-cigarettes and hookahs are especially appealing to adolescents and young people, particularly those who have not started to smoke^8^. These products are perceived as safer, less harmful and less addictive than cigarettes^32,33,35^ and are more socially acceptable^9,33^. This lower perception of risk, the pressure of the social context in which this consumption occurs, and the greater possibility of offers of tobacco consumption can increase individuals’ susceptibility to smoking nicotine products that generate dependence^36–38^. Several studies have indicated that e-cigarettes may be a gateway to cigarette smoking^8,39,40^. Furthermore, the portability problems of the hookah can also lead users to begin smoking cigarettes^33^.

Some authors suggest that the increasing appeal of hookahs and e-cigarettes could slow or reverse the progress achieved in the fight against smoking^32,41^. Since some young people have not yet started smoking but are exposed to smoking devices that are attractive and are perceived as less risky than tobacco, it is important to know the ESSI scores associated with the different devices and to analyse the factors that are associated with a higher ESSI to develop and adapt educational prevention programmes for this new reality.

## Methodology

### Population and sample

#### Inclusion and exclusion criteria

The reference population for this study was students in grades 2 to 4 of compulsory secondary education (aged 12 to 16 years) in the city of Cáceres, Spain. Students who did not obtain parental consent and those who declined to participate in the study even with parental consent were excluded.

#### Sample size calculation

The number of students enrolled in educational centres of Cáceres in the academic year 2017/2018 was n = 11,063^42^. Considering this population size and taking into account that the prevalence of susceptibility to tobacco products in non-smokers found in previous studies was approximately 25%^22,26,28^, we estimated that a random sample of a minimum of 296 students would be sufficient, with a 95% confidence level, 5% margin of error and 5% replacement rate. The formula to calculate the sample size “n” was n = N*X/(X + N – 1), where X = Zα/2^2^ − *p*(1 − p)/MOE^2^. “Zα/2” is the critical value of the normal distribution at α/2 (e.g., for a confidence level of 95%, α is 0.05 and the critical value is 1.96), “MOE” is the margin of error, “p” is the sample proportion, and “N” is the population size. Finite population correction was applied to the sample size formula, and a replacement rate of 5% was anticipated^43^.

### Procedure

The student sample was collected from November 2019 to March 2020 from three schools selected randomly from all public schools in the city. The city of Cáceres has a public network of 19 schools that cover 100% of school-aged children (compulsory schooling is up to the age of 16 in Spain). Once authorization was obtained from the selected educational centre, all students enrolled in the grades specified above were invited to participate. Parental written informed consent was requested from the parents or legal guardians of the students by sending letters. Students for whom consent was obtained were given a computer-aided self-administered questionnaire. The students completed the questionnaire voluntarily, and a project researcher was present at all times to provide assistance and resolve concerns. This study was approved by the Institutional Review Board of the University of Extremadura (Cod. 187/2019) and adhered to the tenets of the Declaration of Helsinki.

### Data collection instrument

Data collection was conducted using a computer-aided self-administered questionnaire. This anonymized questionnaire collected data on socioeconomic variables and a battery of validated scales. The scales and the references for validation in the population are presented in the following sections. Before the study began, a pilot test was conducted with 25 volunteer students to detect difficulties in the execution of the study. In all cases, the students were provided with detailed information in the questionnaire, and a researcher was present in the classroom to answer questions. No incidents were reported during the pilot test or the data collection period of the study.

### Measurements

#### Main study variable

The ESSI values for three products—cigarettes, e-cigarettes and hookahs—were evaluated by measuring SS and curiosity. SS was measured using three questions developed by Pierce et al.^21^ and adapted to each of the products: (i) “Do you think that you will try a (cigarette/e-cigarette/hookah) soon?”, (ii) “Do you think you will smoke a (cigarette/e-cigarette/hookah) in the next year?” and (iii) “If one of your best friends were to offer you a (cigarette/e-cigarette/hookah), would you smoke it?” The four response options were “Definitely not”, “Probably not”, “Probably yes” and “Definitely yes”. The participants who answered “Definitely not” to the three questions were classified as not susceptible to smoking. Those who answered “Probably yes” or “Definitely yes” to any question were classified as highly susceptible. Those who did not meet these descriptions were classified as susceptible^23,36,44,45^. This score had an internal reliability (Cronbach’s α) of 0.72^18^. This scale has proven to be a valid predictor of future smoking initiation^21^.

Curiosity was assessed for each product using a validated question^18^: “Have you ever been curious about smoking a (cigarette/e-cigarette/hookah)?” The possible responses were “Definitely not”, “Probably not”, “Probably yes” and “Definitely yes”. Participants who answered “Definitely not” were classified as not curious, those who answered “Probably not” and “Probably yes” were classified as curious, and those who answered “Definitely yes” were classified as highly curious. This variable has been shown to be a predictor of initiation^18,23^.

The expanded susceptibility index was obtained by adding the SS and curiosity indices, which yielded groups with scores from 0 to 4. The original index assigned a value of 0 to those classified as not susceptible, 1 to those classified as susceptible and 2 to those classified as highly susceptible; similar values were assigned for curiosity. All scores for SS and curiosity were summed to yield an individual’s susceptibility index. This index has an internal reliability of 0.74 (Cronbach’s α)^18^ and has been shown to be a good predictor of the initiation of smoking among adolescents^19^.

#### Independent variables

The following variables were included: age (years), gender (male/female/prefer not to say), household composition, family members with whom the student lives all or most of the time, and school year. Students who lived with their parents were asked about the level of education of their mother and father using a graduated scale with four levels: "Never studied”, “Primary studies", “Secondary studies” and "University studies"^46^. Socioeconomic status was assessed using the Family Affluence Scale (FASII), which assesses family wealth^47^ and has been used in other studies in our context^46^. The FASII scale consists of 4 items with several response options. Each response is assigned a value. The scores are added, and the value obtained is used to classify family affluence as low (0–2 points), average (3–5 points) or high (6–9 points).

The use of the different products was evaluated using two questions that assessed different degrees of use: “Have you ever tried or experimented with (cigarette/e-cigarette/hookah) smoking, even a few puffs?”^48^ If the response was “yes”, a second question was asked: “Have you ever smoked a (cigarette/e-cigarette/hookah)? Do not answer ‘yes’ if you only took a few puffs of someone else’s (cigarette/e-cigarette/hookah)”^49^. Among smokers, the age at initiation/first experimentation with each product and the frequency of use in the previous 30 days were determined^46^. Risk perceptions related to the likelihood of developing health issues as a result of using each product were also determined, with possible responses of “It will not happen”, “Not likely”, “Likely”, “Very likely”, “It will definitely happen” and “I do not know”^9^.

The use of these products in the adolescents’ social and family environment was evaluated with questions about the use habits of each member of the family unit as well as peers (classmates), friends and the respondent’s 5 best friends. We asked about the use of cigarettes/e-cigarettes/hookahs among each group, and the responses were classified into five grades (from 1 = “Almost everyone” to 5 = “Almost no one”)^32^. The frequency of exposure to use was assessed by asking the student about exposure during the last 7 days. Offers to smoke in the last 30 days were also assessed^28^.

Information about alcohol and other drug use in the previous 30 days was collected through the timeline follow-back (TLFB) method^50^. To establish whether alcohol use was problematic, we used the Alcohol Use Disorders Identification Test-Consumption (AUDIT-C), which has shown good internal consistency in the adolescent population (Cronbach’s α of 0.82)^51^.

Impulsivity was assessed using the UPPS-P impulsive behaviour scale^52^. This scale evaluates four factors of impulsivity (urgency, sensation seeking, lack of perseverance and lack of premeditation) through 20 items with a 5-point Likert-type response option. The scale has been validated in the Spanish population and has an internal reliability (Cronbach’s α) greater than 0.7^53^. The Positive and Negative Affect Scale (PANAS) was used to assess positive affect (PA) and negative affect (NA). The scale consists of 20 items, with 10 for each type of affect. In the adolescent population, the questionnaire presents an internal reliability of Cronbach’s α = 0.74 for boys and 0.75 for girls for the NA scale and 0.73 for boys and 0.72 for girls for the PA scale^54^.

### Statistical analysis

Descriptive analyses were performed to study the distribution of the variables and the presence of outliers. The normality of the distribution of the quantitative variables was verified using measures of central tendency and dispersion, the mean and standard deviation (± SD) when the data had a normal distribution, and the median and interquartile range [IQR] when they did not. Quantitative variables were compared between groups by means of Student’s t-test for variables with normal distribution and with the Kruskal–Wallis test for variables with non-normal distribution. The Pearson chi-square test was used to compare categorical variables. The multivariate analysis of the SS was conducted using two models. First, binomial logistic regression was performed with cigarette use in the previous 30 days as a dependent variable and the remaining variables as independent variables. The second model used multinomial logistic regression to analyse students who had never tried cigarettes by considering the ESSI results (categorized into three levels: not susceptible, susceptible and highly susceptible) as a dependent variable and the rest of the variables as independent variables. This yielded an adjusted odds ratio (ORa) and its corresponding 95% confidence intervals. The analyses were performed using SPSS 24.0 for Windows (SPSS, Chicago, IL).

## Results

Of the 488 students who were invited to participate in the study, consent was obtained from parents or legal guardians for 436 (89.3%). Of these, 19 (4.4%) declined to participate, 16 (3.7%) did not complete the questionnaire, and 24 (5.5%) were removed from the analysis for inconsistencies in their responses. Of the final sample of 377 students, the median age in years was 15 [14–15]. Of the 364 students who answered the gender question, 171 (47%) reported being male. The vast majority lived with their mother 362 (96.0%) and/or father 328 (87%) and siblings 301 (79.6%). Less than half of the students surveyed lived with a parent with a university education (mother 42%, father 36%). Only 11 students (2.9%) reported having low family purchasing power (Table 1).

**Table 1:** Distribution of variables for the entire sample and according to cigarette use.

	Total (n = 377)	Smokers (n = 77)	Non-smokers (n = 300)	p-value
**Gender (N = 364) n (%)**				
Male	171 (47.0)	21 (28.0)	150 (51.9)	< 0.001
Female	193 (53.0)	54 (72.0)	139 (48.1)	
**Age median [IQR]**	15 [14–15]	15 [15–16]	14 [13–15]	< 0.001
**Household composition n (%)**				
Mother	362 (96.1)	74 (96.1)	288 (96.0)	0.967
Father	328 (87.0)	64 (83.1)	264 (88.0)	0.256
Mother or father	369 (97.9)	76 (98.7)	293 (97.7)	0.574
Siblings	301 (79.6)	67 (87.0)	234 (78.0)	0.079
Grandparents	147 (39.0)	36 (46.8)	111 (37.0)	0.118
Other cohabitants	97 (25.7)	26 (33.8)	71 (23.7)	0.051
**Maternal education (N = 362) n (%)**				
University	152 (42.0)	26 (35.1)	126 (43.8)	
Secondary	127 (35.1)	24 (32.4)	103 (35.8)	0.222
Primary/none	83 (22.9)	24 (32.4)	59 (20.5)	
**Paternal education (N = 328) n (%)**				
University	118 (36.0)	19 (29.7)	99 (37.5)	
Secondary	114 (34.8)	21 (32.8)	93 (35.2)	0.306
Primary/none	96 (29.3)	24 (37.5)	72 (27.3)	
**Socioeconomic status n (%)**				
Low	11 (2.9)	1 (1.3)	10 (3.3)	
Average	133 (35.3)	27 (35.1)	106 (35.3)	0.630
High	233 (61.8)	49 (63.6)	184 (61.3)	
**Use in previous 30 days n (%)**				
E-cigarette	36 (9.5)	22 (28.6)	14 (4.7)	< 0.001
Hookah	56 (14.9)	31 (40.3)	25 (8.3)	< 0.001
Cannabis	23 (6.1)	18 (23.4)	5 (1.7)	< 0.001
Other drugs	10 (2.7)	4 (5.2)	6 (1.8)	0.126
**Alcoholic beverage use n (%)**				
Never	219 (58.1)	9 (11.7)	210 (70.0)	
Once or less a month	96 (25.5)	27 (35.1)	69 (23.0)	< 0.001
2–4 times a month	53 (14.1)	34 (44.2)	19 (6.3)	
2–3 times a week or more	9 (2.4)	7 (9.1)	2 (0.7)	
**Audit-C test n (%)**	53 (14.1)	34 (44.2)	19 (6.3)	< 0.001
**UPPS-P impulsivity scale median [IQR]**				
Urgency	19 [16–23]	22 [16–23]	25 [22–28]	< 0.001
Sensation seeking	13 [10–17.5]	12.5 [9–16]	14 [10–16]	0.660
Lack of perseverance	7 [5.5–9]	8 [6–10]	8 [6–11]	< 0.001
Lack of premeditation	9 [7–12]	9 [7–11]	11 [8.5–13)	< 0.001
Total	49 [43–55.5]	51 [46–58]	58 [52–63]	< 0.001
**PANAS median [IQR]**				
Positive	24 [22–27]	25 [22.5–26]	24 [22–27]	0.829
Negative	20 [17.5–22.5]	21 [19–23]	20 [17–22]	0.004
Total	44 [42–47]	45 [43–48.5]	44 [41–47]	0.011

IQR: interquartile range; UPPS-P: impulsive behaviour scale; PANAS: positive and negative affect scale.

Of the total sample of students, n = 226 (60%) had never tried tobacco, 74 (19.6%) had tried it but had not used it in the last 30 days, and 77 (20.4%) had smoked in the previous 30 days. The median age in years at which the first use occurred was 14 [13–14]. For e-cigarettes, we found that 204 (54.1%) of students had never tried them, 107 (28.4%) had tried them but had not used them in the previous 30 days, and 36 (9.5%) had smoked them in the previous 30 days. Regarding e-cigarettes, the median age at which they were first tried was 13 [13–14] years. Regarding hookahs, we found that 250 (66.3%) of the students had not tried them, 71 (18.8%) had tried them but had not used one in the previous 30 days, and 56 (14.9%) had smoked one in the previous 30 days. The median [IQR] age at the first use of a hookah was 14 [13–14] years.

When comparing students who had and had not smoked in the previous 30 days (Table 1), we found that the majority were female 54 (72%), and the median age in years for the smokers was 15 years [15–16] vs. 14 years for the non-smokers [13–15] (p < 0.001). There was no age difference between the genders (15 [14–15] years for both males and females; p = 0.184). The percentage of smokers who also smoked e-cigarettes or hookahs was significantly higher than that of non-smokers (p < 0.001 for both products). The rate of alcohol consumption was much higher among smokers; only 9 (11.7%) of the smokers reported never having consumed alcohol compared to 210 (70.0%) of the non-smokers (p < 0.001). The Audit-C screening test for alcohol risk was positive in 34 (44.2%) smokers compared to 19 (6.3%) non-smokers (p < 0.001). We also found that a higher percentage of smokers than non-smokers had used cannabis in the previous 30 days (23.4% vs. 1.7%; p < 0.001). Regarding impulsivity, we found significantly higher scores on the UPPS-P scale among smokers, with a total score of 57.9 (± 8.4) for smokers vs. 52.6 (± 9.2) for non-smokers (p < 0.001). The differences in the scores occurred in the dimensions of urgency (p < 0.001), lack of perseverance (p = 0.012) and lack of premeditation (p < 0.001). We also found higher scores on the PANAS among smokers than among non-smokers (p = 0.011), with notable differences in the NA scale scores (p = 0.004).

When the smoking environment of smokers and non-smokers in the previous 30 days was analysed (Table 2), we found that household smoking was higher among smokers than among the non-smokers, and there were significant differences between the groups in the proportions of mothers (p < 0.001), fathers (p < 0.011), siblings (p < 0.001) and other cohabitants (p = 0.037) who smoked. There were also differences in the number of days students were exposed to smoke in the home; most non-smokers, 215 (71.7%), stated that they were not exposed to second-hand smoke any day compared to 37 (48.1%) of the smokers (p < 0.001). These differences were also found in terms of perceptions of smoking in the family environment; almost half, 143 (47.7%), of the non-smoking students reported that almost no one in their family environment smoked, while only 5.2% of the smoking students had the same perception. We also found significant differences in perceptions regarding smoking among peers: 15 (19.5%) of the smokers felt that almost none of their peers smoked, while 114 (38.0%) of non-smokers had that perception (p = 0.012). We found even more significant differences regarding perceptions of cigarette smoking among the respondents’ five best friends (p < 0.001). Regarding the perceived risk of smoking, we found that 209 (69.7%) of the non-smokers indicated a high perceived risk (responding that tobacco use would very likely or definitely have serious health consequences) compared to 34 (44.2%) of the smokers (p < 0.001).

**Table 2:** Cigarette use in the environment and its perceived risk.

	Total (n = 377)	Smokers (n = 77)	Non-smokers (n = 300)	p-value
**Cigarette smoking in the household n (%)**				
Mother (n = 362)	98 (27.1)	35 (47.3)	63 (21.9)	< 0.001
Father (n = 328)	87 (26.5)	25 (39.1)	62 (23.5)	0.011
Siblings (n = 301)	58 (19.3)	24 (35.5)	43 (18.4)	< 0.001
Grandparents (n = 147)	24 (16.3)	7 (19.4)	17 (15.3)	0.363
Other cohabitants (n = 91)	55 (55.7)	19 (73.1)	35 (49.3)	0.037
**Exposure to second-hand smoke in the home (in the previous 7 days) n (%)**				
None	252 (66.8)	37 (48.1)	215 (71.7)	
1–2 days	31 (8.2)	7 (9.1)	24 (8.0)	
3–4 days	18 (4.8)	7 (9.1)	11 (3.7)	< 0.001
5–6 days	10 (2.7)	3 (3.9)	7 (2.3)	
All 7 days	66 (17.5)	23 (29.9)	43 (14.3)	
**Perception of household smoking n (%)**				
Almost no one	147 (39)	4 (5.2)	143 (47.7)	
Less than half	72 (19.1)	10 (13.0)	62 (20.7)	
Half	38 (10.1)	11 (14.3)	27 (9.0)	< 0.001
More than half	59 (15.6)	18 (23.4)	41 (13.7)	
Almost everyone	61 (16.2)	34 (44.2)	27 (9.0)	
**Perception of peer smoking n (%)**				
Almost no one	129 (34.2)	15 (19.5)	114 (38.0)	
Less than half	154 (40.8)	33 (42.9)	121 (40.3)	
Half	52 (13.8)	15 (19.5)	37 (12.3)	0.012
More than half	31 (8.2)	10 (13)	21 (7.0)	
Almost everyone	11 (2.9)	4 (5.2)	7 (2.3)	
**Smoking among the five best friends n (%)**				
No one	170 (45.1)	5 (6.5)	165 (55.0)	
1–2	85 (22.6)	8 (10.4)	77 (25.7)	< 0.001
3–4	86 (22.9)	42 (54.6)	44 (14.7)	
Everyone	36 (9.5)	22 (28.6)	14 (4.7)	
**Perceived risk from smoking n (%)**				
Will not happen	28 (7.4)	3 (3.9)	25 (8.3)	
Not likely	17 (4.5)	7 (9.1)	10 (3.3)	
Likely	57 (15.1)	23 (29.9)	34 (11.3)	< 0.001
Very likely	166 (44.0)	26 (33.8)	140 (46.7)	
Will definitely happen	77 (20.4)	8 (10.4)	69 (23.0)	
Don’t know	32 (8.5)	10 (12.0)	22 (7.3)	

**Figure 1:**
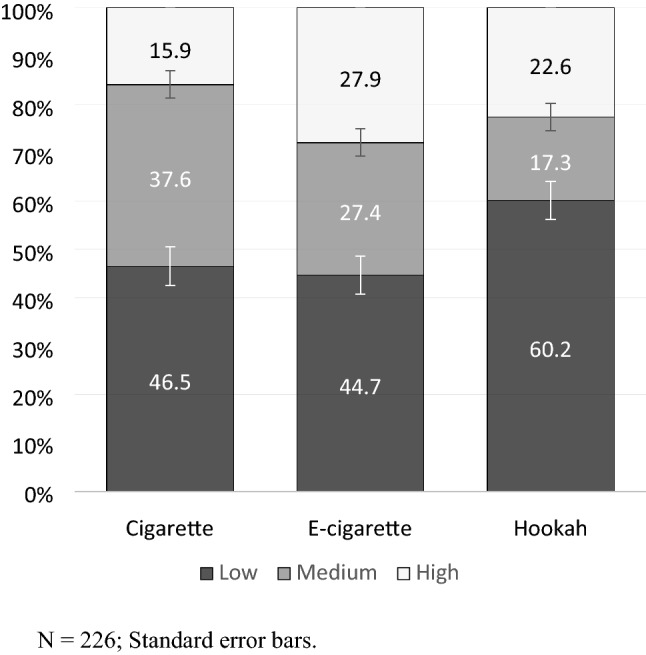
Expanded Susceptibility to Smoking Index (ESSI) for non-smoking students. N = 226; Standard error bars.

When analysing susceptibility to smoking among students who had never smoked (n = 226), we found that 80 (35.4%) had a medium or high SS for cigarette smoking, 110 (48.7%) had a medium or high SS for e-cigarettes, and 87 (38.4%) had a medium or high SS for hookah smoking. Eighty-one (36.0%) were curious about cigarettes, 100 (43.8%) about e-cigarettes and 65 (28.8%) about hookahs. When both indicators in the ESSI were combined, we found that 36 (15.9%) students were highly susceptible to cigarette smoking, 63 (27.9%) were highly susceptible to e-cigarette smoking, and 51 (22.6%) were highly susceptible to hookah smoking (Fig. 1).

When comparing the distribution of the variables according to the ESSI in students who had not tried cigarettes (Table 3), we found that there was a significantly lower presence of both parents in the home (p = 0.042) and a greater presence of grandparents (p = 0.017) among students classified as highly susceptible. In the high-susceptibility group, a significantly higher percentage of students (16, 44.4%) had experimented with e-cigarettes than in the susceptible (23, 27.1%) and non-susceptible (10, 9.5%) groups, p < 0.001. There were also differences among the groups in the frequency of alcohol consumption: 7 (6.7%) students in the non-susceptible group reported consuming alcohol at least once a month compared with 13 (36.1%) of the students in the highly susceptible group (p < 0.001). Furthermore, 3 (8.3%) students in the highly susceptible group were considered at high risk of alcohol abuse according to the Audit-C (p = 0.022). The UPPS-P showed higher impulsivity scores among students who were highly susceptible to smoking (p < 0.001). This difference was due to higher scores for urgency (< 0.001) and lack of perseverance (p = 0.039) among the highly susceptible students.

**Table 3:** Comparison of variables according to the Expanded Susceptibility to Smoking Index (ESSI) values for students who had not tried cigarettes.

	ESSI			p-value
	Not susceptible (n = 105)	Susceptible (n = 85)	Highly Susceptible (n = 36)	
**Age (years) median [IQR]**	14 [13–15]	14 [13–15]	14 [13–15]	0.490
**Gender male n (%)**	63 (61.8)	43 (50.6)	14 (41.2)	0.077
**Household composition n (%)**				
Mother	104 (99.0)	82 (96.5)	33 (91.7)	0.084
Father	94 (89.5)	77 (90.6)	30 (83.3)	0.491
Mother or father	105 (100)	84 (98.8)	34 (94.4)	0.042
Siblings	90 (85.7)	61 (71.8)	28 (77.8)	0.061
Grandparents	38 (36.2)	22 (25.9)	19 (52.8)	0.017
Other cohabitants	21 (20.0)	19 (22.4)	10 (27.89	0.623
**Maternal education (N = 219) n (%)**				
University	46 (43.2)	44 (53.7)	13 (39.4)	
Secondary	40 (38.5)	23 (28.0)	12 (36.4)	0.191
Primary/none	18 (17.3)	15 (18.3)	8 (24.2)	
**Paternal education (N = 201) n (%)**				
University	35 (37.2)	38 (49.4)	10 (33.3)	
Secondary	31 (28.7)	23 (29.9)	11 (36.7)	0.572
Primary/none	28 (29.8)	16 (20.8)	9 (30.0)	
**Socioeconomic status n (%)**				
Low	5 (4.8)	2 (2.4)	2 (5.6)	
Average	30 (28.6)	29 (34.1)	12 (33.3)	0.806
High	70 (66.5)	54 (63.5)	22 (61.1)	
**Tried n (%)**				
E-cigarette	10 (9.5)	23 (27.1)	16 (44.4)	< 0.001
Hookah	19 (18.1)	14 (16.5)	6 (16.7)	0.953
Other drugs	4 (4.0)	4 (4.8)	0 (0.0)	0.442
**Alcoholic beverage use n (%)**				
Never	98 (93.3)	67 (78.8)	23 (63.9)	
Once or less a month	6 (5.7)	17 (20.0)	11 (30.5)	0.001
Two to four times a month or more	1 (1.0)	1 (1.2)	2 (5.6%)	
**Audit-C test n (%)**	0 (0.0)	3 (3.5)	3 (8.3)	0.022
**UPPS-P impulsivity scale median [IQR]**				
Urgency	19 [16–23]	22 [19–25]	25 [22.5–27.5]	< 0.001
Sensation seeking	13 [10–16]	13 [10–16]	14 [10–16]	0.918
Lack of perseverance	7 [6–9]	8 [6–9]	8 [6.5–11]	0.039
Lack of premeditation	9 [7–12]	9 [7–11]	10.5 [8–13]	0.070
Total	49 [43–55]	51 [46–58]	59 [50.5–62.5]	< 0.001
**PANAS median [IQR]**				
Positive affect	25 [23–27]	25 [22–26.5]	24 [23–26]	0.293
Negative affect	19 [17–21]	19 [17–22]	20.5 [17–23]	0.288
Total	44 [41–46]	44 [40.5–47]	44.5 [41–47]	0.796

IQR: interquartile range; UPPS-P: impulsive behaviour scale; PANAS: positive and negative affect scale.

When the smoking environment and perceived risk were compared among the different susceptibility groups (Table 4), we found higher percentages of mothers and siblings who smoked among highly susceptible students at 13 (39.4%) (p = 0.019) and 6 (21.4%) (p = 0.013), respectively. Differences were also found in the perception of household smoking (p = 0.002). For the rest of the variables, we did not find significant differences except in the percentage of students who received offers to smoke (p = 0.007).

**Table 4:** Smoking environment and perceived risk according to the Expanded Susceptibility to Smoking Index (ESSI) in students who had not tried cigarettes.

	ESSI			p-value
	Not susceptible (n = 105)	Susceptible (n = 85)	Highly Susceptible (n = 36)	
**Smoking in the household n (%)**				
Mother (n = 219)	19 (18.3)	14 (17.1)	13 (39.4)	0.019
Father (n = 201)	20 (21.3)	16 (20.8)	10 (33.3)	0.335
Siblings (n = 179)	4 (4.4)	10 (16.4)	6 (21.4)	0.013
Grandparents (n = 79)	3 (7.9)	3 (13.6)	6 (31.6)	0.062
Other cohabitants (n = 50)	8 (38.1)	8 (42.1)	7 (70.0)	0.227
**Exposure to second-hand smoke in the home (previous 7 days) n (%)**				
None	82 (78.1)	65 (76.5)	20 (55.6)	
1–2 days	8 (7.6)	4 (4.7)	7 (19.4)	
3–4 days	2 (1.9)	2 (2.4)	0 (0.0)	0.075
5–6 days	2 (1.9)	0 (0.0)	1 (2.8)	
All 7 days	11 (10.5)	14 (16.5)	8 (22.2)	
**Perception of household smoking n (%)**				
Almost no one	72 (68.6)	45 (52.9)	13 (36.1)	
Less than half	17 (16.2)	19 (22.4)	11 (30.6)	
Half	4 (3.8)	6 (7.1)	6 (16.7)	0.002
More than half	6 (5.7)	10 (11.8)	4 (11.1)	
Almost everyone	6 (5.7)	5 (5.9)	2 (5.6)	
**Perception of peer smoking n (%)**				
Almost no one	44 (41.9)	35 (41.2)	12 (33.3)	
Less than half	42 (40.0)	36 (42.2)	16 (44.4)	
Half	19 (9.5)	7 (8.2)	7 (19.4)	0.458
More than half	8 (7.6)	4 (4.7)	1 (2.8)	
Almost everyone	1 (1.0)	3 (3.5)	0 (0.0)	
**Smoking among five best friends n (%)**				
None	79 (75.2)	51 (60)	20 (55.6)	0.083
1–2	17 (16.2)	21 (24.7)	14 (38.8)	
3–4	6 (5.7)	10(11.8)	2 (5.6)	
Everyone	3 (2.9)	3 (3.5)	0 (0.0)	
**Perceived risk from smoking n (%)**				
Will not happen	15 (14.3)	4 (4.7)	2 (5.6)	
Not likely	2 (1.9)	2 (2.4)	3 (8.3)	
Likely	8 (7.6)	12 (14.1)	5 (13.9)	0.140
Very likely	47 (44.8)	48 (56.5)	16 (44.4)	
Will definitely happen	27 (25.7)	15 (17.6)	7 (19.4)	
Don’t know	6 (5.7)	4 (4.7)	3 (8.3)	
**Offers to smoke n (%)**	13 (12.4)	19 (22.4)	13 (36.1)	0.007

The results of the first multivariate analysis model (Table 5) showed a strong association between cigarette use and older age, aOR: 1.79 (95% CI 1.28–2.49) p < 0.001, and, to a lesser extent, female gender, aOR: 2.29 (95% CI 1.03–5.13) p = 0.043. The presence of friends who smoked and the perception that half or more than half of the student’s peers smoked cigarettes were associated with smoking, with an aOR of 6.19 (95% CI 1.72–22.32) p = 0.005 and 2.46 (95% CI 1.03–5.88) p = 0.043, respectively. Significant associations were also found between cigarette smoking and the consumption of alcohol, aOR: 1.79 (95% CI 1.17–4.65) p = 0.035, and other drugs, aOR: 8.26 (95% CI 2.41–28.36) p < 0.001. E-cigarette use was also associated with cigarette smoking (aOR: 4.33 [95% CI 1.45–12.9] p = 0.009). On the impulsivity scale (UPPS-P), lack of premeditation was the best predictor of cigarette use (aOR: 1.15 [95% CI 1.01–1.32] p = 0.035), and on the PANAS, the PA scale had the highest association with cigarette use (aOR: 1.16 [95% CI 1.02–1.32] p = 0.029).

**Table 5:** Multivariate analysis 1.

Exposure	aOR	95% CI	p-value
**Age**			
1-year increase	**1.79**	**1.28–2.49**	** < 0.001**
**Gender**			
Male	1.00 Ref		
Female	**2.29**	**1.03–5.13**	**0.043**
**Socioeconomic status**			
Medium or high	1.00 Ref		
Low	1.14	0.54–2.39	0.726
**Household exposure**			
No	1.00 Ref		
Yes	2.02	0.51–7.98	0.316
**Friends who smoke**			
No	1.00 Ref		
Yes	**6.19**	**1.72–22.32**	**0.005**
**Perception of peer smoking**			
Less than half	1.00 Ref		
Half or more	**2.46**	**1.03–5.88**	**0.043**
**Alcohol consumption**			
No	1.00 Ref		
Yes	**1.79**	**1.17–4.65**	**0.035**
**Use of other drugs**			
No	1.00 Ref		
Yes	**8.26**	**2.41–28.36**	** < 0.001**
**E-cigarette use**			
No	1.00 Ref		
Yes	**4.33**	**1.45–12.9**	**0.009**
**Hookah use**			
No	1.00 Ref		
Yes	2.23	0.91–5.45	0.080
**Perceived risk from smoking**			
Will not happen/not likely	1.00 Ref		
Likely to happen to definitely will happen	0.59	0.25–1.38	0.222
**UPPS-P (1-point increase)**			
Urgency	1.04	0.96–1.14	0.342
Sensation seeking	1.00	0.92–1.09	0.921
Lack of perseverance	1.01	0.86–1.18	0.905
Lack of premeditation	**1.15**	**1.01–1.32**	**0.035**
**PANAS (1-point increase)**			
Positive affect	**1.16**	**1.02–1.32**	**0.029**
Negative affect	1.04	0.93–1.17	0.497

n = 377.

Significant variables and results shown in bold text.

aOR: adjusted odds ratio; 95% CI: 95% confidence interval; Ref.: reference; UPPS-P: impulsive behaviour scale; PANAS: positive and negative affect scale.

When we analysed the association of the different variables with the ESSI using model 2 (Table 6), we found that exposure to household smoking and the presence of friends who smoked was associated with high susceptibility to smoking (aOR: 2.78 (95% CI 1.17–6.51), p = 0.020 and 3.85 (95% CI 1.67–9.04), p = 0.002, respectively). Alcohol consumption was also strongly associated with being classified as highly susceptible (aOR: 4.67 [95% CI 1.74–12.50], p = 0.002). The perception that smoking cigarettes was likely to have an effect on health was inversely associated with smoking (aOR: 0.35 [95% CI 0.14–0.89], p = 0.028). However, this association was not found for the highly susceptible group. The urgency subscale of the impulsivity scale yielded highly significant associations for both the susceptible and highly susceptible groups, with aORs of 1.15 (95% CI 1.06–1.25), p < 0.001 and 1.17 (95% CI 1.05–1.31), p = 0.005, respectively. Regarding the other subsections of the impulsivity scale, we found an inverse association between lack of perseverance and susceptibility (aOR: 0.86 [95% CI 0.76–0, 98], p = 0.024) that did not occur for highly susceptible students and a positive association between lack of perseverance and high susceptibility to smoking (aOR: 1.22 [95% CI 1.01–1.49], p = 0.044).

**Table 6:** Multivariate analysis 2.

Exposure	ESSI					
	Susceptible			Highly susceptible		
	aOR	95% CI	p-value	aOR	95% CI	p-value
**Age**						
1-year increase	1.01	0.74–1.39	0.926	1.56	0.97–2.48	0.065
**Gender**						
Male	1.00 Ref			1.00 Ref		
Female	1.52	0.77–2.95	0.226	1.75	0.67–4.53	0.252
**Socioeconomic status**						
Medium or high	1.00 Ref			1.00 Ref		
Low	1.12	0.56–2.22	0.749	0.93	0.36–2.39	0.868
**Household exposure**						
No	1.00 Ref			1.00 Ref		
Yes	1.39	0.65–3.00	0.400	**2.78**	**1.17–6.51**	**0.020**
**Smoking friends**						
No	1.00 Ref			1.00 Ref		
Yes	1.75	0.83–3.73	0.137	**3.85**	**1.67–9.04**	**0.002**
**Perception of peer smoking**						
Less than half	1.00 Ref			1.00 Ref		
Half or more	0.83	0.45–1.55	0.560	1.45	0.69–3.04	0.329
**Alcohol consumption**						
No	1.00 Ref			1.00 Ref		
Yes	1.94	0.69–5.39	0.207	**4.67**	**1.74–12.50**	**0.002**
**Use of other drugs**						
No	1.00 Ref			1.00 Ref		
Yes	3.23	0.24–41.66	0.377	9.09	0.73–104.72	0.088
**E-cigarette use**						
No	1.00 Ref			1.00 Ref		
Yes	2.33	0.12–43.36	0.575	3.45	0.16–75.31	0.424
**Hookah use**						
No	1.00 Ref			1.00 Ref		
Yes	0.61	0.15–2.49	0.491	0.57	0.08–4.38	0.592
**Perceived risk from smoking**						
Will not happen/not likely	1.00 Ref			1.00 Ref		
Likely to happen to definitely will happen	**0.35**	**0.14–0.89**	**0.028**	0.92	0.31–2.74	0.883
**UPPS-P (1-point increase)**						
Urgency	**1.15**	**1.06–1.25**	** < 0.001**	**1.17**	**1.05–1.31**	**0.005**
Sensation seeking	0.98	0.9–1.06	0.558	0.96	0.86–1.07	0.458
Lack of perseverance	1.08	0.93–1.26	0.320	**1.22**	**1.01–1.49**	**0.044**
Lack of premeditation	**0.86**	**0.76–0.98**	**0.024**	0.9	0.75–1.08	0.274
**PANAS (1-point increase)**						
Positive affect	0.95	0.84–1.08	0.446	0.99	0.83–1.18	0.916
Negative affect	0.93	0.84–1.03	0.173	0.98	0.85–1.14	0.799

n = 226.

Significant variables and results shown in bold text.

aOR: adjusted odds ratio; 95% CI: 95% confidence interval; Ref.: reference; ESSI: expanded susceptibility to smoking index; UPPS-P: impulsive behaviour scale; PANAS: positive and negative affect scale.

## Discussion

The results of this study show that a significant number of students between 12 and 16 years of age who have not tried tobacco have some degree of susceptibility to smoking. More than 50% had ESSI values consistent with medium or high susceptibility to cigarette and e-cigarette smoking, and almost 40% had ESSI values indicative of medium or high susceptibility to hookah smoking. It is noteworthy that a greater proportion of students had high ESSI scores for e-cigarette and hookah products (27.9% and 22.6%) than for cigarettes (15.9%), suggesting that these products could be a possible gateway to smoking, as several studies have suggested^30,39^.

Based on the SS component of the ESSI, we found that one of every two non-smoking students was susceptible to smoking e-cigarettes (48.7%), followed by hookahs and cigarettes (38.4% and 35.4%, respectively). The other component of the ESSI, curiosity, indicated that 43.8% of the students showed curiosity about e-cigarettes, with lower values for cigarettes and hookahs (36.7% and 28.8%, respectively). In this line of research in other countries, higher rates of both susceptibility and curiosity have been found for e-cigarettes than for cigarettes and hookahs^22,55^. The SS identifies adolescents at risk of initiating smoking, but if we consider the progression of smoking habits, several longitudinal studies have reported that half of all susceptible adolescents began smoking during follow-up (1–3 years)^19,44^, with similar values for e-cigarettes and hookahs^44^. Other studies have reported lower rates^22^, although this may be due to the different age ranges of the participants and methods of assessing SS. Taking this evidence as a reference and considering that one in three participants was susceptible to smoking, the number of non-smokers who could start smoking is high. Curiosity has been associated with experimentation and subsequent smoking^18^, and recent research conducted in the adolescent population found that 56% of curious adolescents and 65% of highly curious adolescents began experimentation during follow-up^23^. Taking this evidence into account, the number of non-smoking adolescents who may smoke in the future is likely to increase as a result of curiosity. Curiosity is a frequently reported reason for starting smoking^19,23^.

The combination of SS and curiosity in the ESSI improved its sensitivity for predicting experimentation and use among susceptible non-smoking adolescents compared to the use of SS alone^19,23^. The ESSI identified two-thirds of those who initiated smoking^19^ versus the 50% identified by the SS. The extrapolation of these figures to our results, in which we found that 50% of non-smokers were susceptible to cigarette smoking according to the ESSI, leads us to believe that a large number of students could start smoking during high school, which would significantly increase the number of adolescents who smoke by the end of this educational stage.

We found that 20.4% of the participants were current cigarette smokers, 9.5% smoked e-cigarettes, and 14.9% smoked hookahs. Their first use of these products occurred between 13 and 14 years of age, with earlier initiation for e-cigarettes. Current smokers were more likely to be female, to use other tobacco products along with alcohol and other substances (cannabis), to have friends who smoked and to perceive that more of their friends were smokers. Additionally, current smokers presented higher levels of lack of premeditation and reported greater positive affect. More than half of the non-smokers had moderate or high scores on the susceptibility index. Highly susceptible non-smokers had greater exposure to second-hand smoke in the home, had a greater number of friends who smoked and reported consuming alcohol. They obtained higher scores for urgency and lack of perseverance.

The prevalence of current cigarette and e-cigarette users was 20.4% and 9.5%, respectively. The 2018 Spanish national survey reported a 26.7% prevalence of cigarette use and a 14.9% prevalence of e-cigarette use among adolescents between 14 and 18 years of age^3^. The observed difference may be because our study included a younger population group. The prevalence of hookah use was 14.9%, lower than that in a study of Spanish students that reported a 33.7% prevalence of hookah use^56^. These differences were likely to have occurred because the survey sample included students in higher education. Furthermore, the fact that our study was conducted in three educational centres in a single region, while the Spanish national survey was conducted throughout the country, could partly explain these differences.

Tobacco use during adolescence is influenced by sociodemographic, social and intrapersonal factors. Regarding sociodemographic factors, we found an association between age and smoking that is consistent with other studies^57,58^. Regarding gender, we found a higher prevalence of smoking among females. Although the available evidence reports higher use among female adolescents, this situation is not new, as shown by the Health Behaviour in School-Aged Children (HBSC) study for Spain and the ESTUDES Survey (National Survey on Drugs in Secondary Education)^2,3^. According to ESTUDES, in 2010, the prevalence of smoking among girls aged 14–18 years was 13.6% vs. 11% for boys, and the 2018 data continue to reflect this behaviour, although the difference has narrowed (10.3% for girls vs. 9.4% for boys)^3^.

Within the social environment, we highlight two findings. First, we found an association between exposure to second-hand smoke and highly susceptible non-smoking status. According to the data from a review, the smoking status of household members is a predictor of smoking initiation in adolescents^27^, and its influence may be mediated by exposure to second-hand smoke^59^. It has also been suggested that the influence of smoking among household members is lower than that exerted by the peer group^60^, which was our second finding. Tobacco use within the peer group was associated with both high susceptibility and smoking status. These data seem to be in line with other research that suggests that engaging in risky behaviours during adolescence, such as smoking, seems to be influenced by the peer group^61,62^, which is a key factor in both intentionality and smoking^35^. Some studies suggest that having one or more friends who smoke increases the risk of smoking^57,63^. If smoking among friends occurs in situations in which positive affect prevails, non-smokers might find it difficult to resist peer pressure^64^, which could increase the probability that they will experiment.

Tobacco use has also been linked to other risk behaviours, including the use of alcohol and other substances, such as cannabis^65,66^. We found an association of the combination of tobacco use and high susceptibility to smoking with alcohol consumption, while cannabis use was only associated with smoking status. Alcohol consumption among young people usually occurs in a context where the ease of access to cigarettes is greater^66^. If this situation occurs during states of positive affect, peer pressure could be increased and could be more difficult to resist^64^, which could lead adolescents to experiment with cigarettes.

Regarding intrapersonal factors, previous studies have established the existence of a close relationship between addictive behaviours, impulsivity and personal traits^31^. We found an association of high susceptibility with urgency and lack of perseverance, and current smoking was associated with lack of premeditation and positive affect. According to one review, impulsivity factors are specifically associated with different stages of smoking in the adult population^67^, and smoking increases in cases of global impulsivity. In addition, this relationship is not static and can change over time^67^. In line with our results, other studies have reported that a lack of premeditation and impulsivity based on emotion or positive affect seem to be traits that differentiate smokers from non-smokers^31^. Adolescence is associated with an increase in mood swings; when adolescents are under great emotional burden, they tend to act without thinking and without evaluating the consequences^64^. Hasty action or urgency in situations of intense emotions yields immediate feedback, which could affect the probability that the action will be repeated in the future^31^. Evidence suggests that urgency has a good predictive capacity in relation to risk-taking and substance use^68^. Measures of urgency during primary school seem to predict tobacco use later in life^68,69^.

There are several limitations of this study, including the design; this is a cross-sectional study that reports the existence of an association between the variables studied but does not establish causality in this relationship. Although the selection of the educational centres for the study was performed randomly, the selection of the sample does not depict probability sampling, which could affect the external validity of the results. Furthermore, the data were obtained through self-report, which could influence their veracity, although this approach has been considered adequate to assess consumer behaviour in educational environments^25^. Anonymity and confidentiality were used to improve data veracity. Another limitation is the study population. This study was conducted among students in the 2nd to 4th grades of secondary education who resided in a region of Spain. Consequently, the results obtained may not be generalizable to other areas, although they allow factors related to tobacco use before and after the initiation of smoking to be determined.

The data of this study suggest that among adolescents, the social environment, smoking among family members and especially among friends, other risky behaviours such as alcohol and cannabis use, and situations involving intense emotions where the individual may be hasty in his or her actions influence the susceptibility to and initiation of smoking. These findings could improve prevention programmes in the educational field by taking into account the influence of the social environment and intrapersonal factors on both smoking susceptibility and smoking initiation. Reducing peer pressure through self-efficacy and improving resolve under conditions of positive affect to reduce urgency could be suitable approaches. The findings should guide future longitudinal studies that can confirm the associations detected and propose more effective interventions.
